# ENmix: a novel background correction method for Illumina HumanMethylation450 BeadChip

**DOI:** 10.1093/nar/gkv907

**Published:** 2015-09-17

**Authors:** Zongli Xu, Liang Niu, Leping Li, Jack A. Taylor

**Affiliations:** 1 Epidemiology Branch, National Institute of Environmental Health Sciences, NIH, Research Triangle Park, NC, USA; 2 Biostatistics & Computational Biology Branch, National Institute of Environmental Health Sciences, NIH, Research Triangle Park, NC, USA; 3 Department of Environmental Health, College of Medicine, University of Cincinnati, Cincinnati, OH, USA; 4 Laboratory of Molecular Carcinogenesis, National Institute of Environmental Health Sciences, NIH, Research Triangle Park, NC, USA

## Abstract

The Illumina HumanMethylation450 BeadChip is increasingly utilized in epigenome-wide association studies, however, this array-based measurement of DNA methylation is subject to measurement variation. Appropriate data preprocessing to remove background noise is important for detecting the small changes that may be associated with disease. We developed a novel background correction method, ENmix, that uses a mixture of exponential and truncated normal distributions to flexibly model signal intensity and uses a truncated normal distribution to model background noise. Depending on data availability, we employ three approaches to estimate background normal distribution parameters using (i) internal chip negative controls, (ii) out-of-band Infinium I probe intensities or (iii) combined methylated and unmethylated intensities. We evaluate ENmix against other available methods for both reproducibility among duplicate samples and accuracy of methylation measurement among laboratory control samples. ENmix out-performed other background correction methods for both these measures and substantially reduced the probe-design type bias between Infinium I and II probes. In reanalysis of existing EWAS data we show that ENmix can identify additional CpGs, and results in smaller *P*-value estimates for previously-validated CpGs. We incorporated the method into R package *ENmix*, which is freely available from Bioconductor website.

## INTRODUCTION

DNA methylation is essential for human normal development and regulation of gene expression, while aberrant methylation has been linked with a number of human diseases (1,2). The advance of DNA methylation arrays in recent years has enabled large-scale epigenome-wide studies at single CpG site resolution. The Illumina Infinium HumanMethylation450 BeadChip (3) is currently the most commonly utilized array providing estimation of methylation level at about half a million individual CpG sites. The array is based on measuring probe hybridization intensity values of bisulfite-converted DNA to estimate the relative abundance of methylated and unmethylated cytosines at selected loci. Like gene expression microarrays, these quantitative measures are sensitive to variations in experimental conditions (4). In addition, the array uses probes with two different chemistries (Infinium I and Infinium II) and two fluorescent dyes (Cy3-green/Cy5-red) introducing further complexity to the resulting data.

A number of data preprocessing methods have been proposed to improve methylation data quality. These include methods for background correction (5,6), dye bias correction (7), inter-array normalization (5) and probe-design bias adjustment (8–11). Several background subtraction methods have been proposed: subtracting 5% percentile (Q5) of the negative controls in each color channel (GenomeStudio Methylation Module v1.8), subtracting the median intensity value of negative control probes (lumi1: R package lumi) or subtracting the mode of methylated probe intensities (lumi2: R package lumi). These background subtraction methods can produce negative values or truncate low intensity signals, and thus may introduce additional bias (5). Pidsley et al. proposed background adjustment instead of background correction: adding the offset between Infinium I and II probe intensity values back to Infinium I probe intensities (5). Triche et al. proposed background estimation using ‘out-of-band’ signal intensities for Infinium I probes followed by adjustment using normal-exponential (noob) or gamma convolution (goob) methods (6). However, both noob and goob methods assume that signal intensities are exponentially distributed, an assumption that may not fit most DNA methylation array data.

Here we report that an exponential-normal mixture distribution can closely approximate the observed distributions of signal intensities. Based on this observation, we introduce a novel model-based background correction method, compare the performance (reproducibility and accuracy) of this method to other commonly-used background subtraction methods and provide a user friendly R package *ENmix* that incorporates the method.

## MATERIALS AND METHODS

### Illumina DNA methylation BeadChip

The Illumina Infinium HumanMethylation450 BeadChip uses bisulfite converted DNA to estimate methylated (M) and unmethylated (U) allele intensity at individual CpG site. The methylation level (Beta value) is calculated as M/(M+U+100), where 100 in the denominator is a constant offset recommended by Illumina to regularize Beta values when both methylated and unmethylated intensities are low. Two different assay chemistries are employed to increase CpG coverage. The Infinium I assay is used for 28% (135 476) of the CpGs on array and has two bead types for each CpG locus: one for the methylated and one for the unmethylated alleles. Signal intensities for both alleles at a locus are scanned on the same color channel (Cy3 green for some loci and Cy5 red for others). For a given type I bead, the intensity data from the unused color channel has been proposed as a means to estimate background, and termed the ‘out-of-band’ (oob) intensity (6). The Infinium II assay is used for 72% (350 036) of the CpGs on the array and uses a single bead type per CpG. It utilizes two different colors to represent the two different alleles. These are assessed via single base extension with guanine (labeled with Cy3) for methylated, or adenine (labeled with Cy5) for unmethylated alleles. The HumanMethylation450K Beadchip has 850 internal control probes to monitor experimental procedures at different steps, including 613 negative control probes to measure background intensity and 186 non-polymorphic control probes that can be used to monitor color channel difference.

### Evaluation data sets

The effect of different background correction methods on reproducibility was assessed using data from 20 pairs of duplicate samples that were part of a previously published study of methylation in 891 infant whole blood samples (12). As part of this study, duplicate samples were located on separate 96 well plates that underwent independent bisulfite conversion, hybridization and array scanning. One sample was excluded due to poor data quality, leaving 19 duplicate pairs (38 samples) for evaluation.

The effect of different background correction methods on measurement accuracy was assessed using data from methylation control mixture samples for this same study (12), where purified human 100% methylated and unmethylated DNA (Zymo Research, Irving CA) were mixed together in different proportions to create laboratory control samples with specific methylation levels: 0%, 5%, 10%, 20%, 40%, 50%, 60%, 80% and 100% methylated Replicates for each methylation level (*n* = 10, 3, 2, 3, 3, 2, 3, 3 and 10, respectively) were independently assayed on different arrays.

To avoid possible impact on evaluations, we excluded 69 075 probes, which include non-specific bind probes, common (MAF > 0.05) SNPs at CpG target regions, probes on sex chromosomes and probes with multimodal methylation distributions identified using *ENmix* R package. We also excluded probes with low quality methylation values where the number of beads was less than 3 or detection *P*-value greater than 0.05.

To demonstrate the effect of ENmix background correction method on epigenome-wide association studies (EWAS), we re-analyzed raw blood DNA methylation data from 889 infants in relation to maternal smoking (12). We preprocessed the data with different methods or combinations of methods: raw data, Q5 background correction, ENmix_oob background correction, ENmix and dye bias correction (ENmixD), ENmix+dye bias correction+quantile normalization (ENmixDQ) and ENmix+dye bias correction+quantile normalization+BMIQ (ENmixDQB). We used a robust linear regression model to test for association between maternal smoking and infant DNA methylation level adjusting for the following variables: cell type proportion (CD8T, CD4T, NK, Bcell, Mono and Gran) estimated using the Houseman method (13) from minfi R package, gestational age in weeks, sex, education in two categories, birth weight, maternal age, maternal BMI, parity, experimental batch, cleft phenotype and baby birth year.

### ENmix: Exponential-Normal mixture signal intensity background correction

Frequency plots of methylation beta values for CpGs on the Illumina 450K array have a characteristic bimodal distribution for many human tissues, such that the majority of CpGs tend to be either unmethylated or highly methylated (Supplementary Figure S1A). Frequency plots of the underlying probe intensity values from which these beta values are calculated have heavy tails to the right for either methylated and unmethylated intensities (Supplementary Figure S1B and S1C). Considering only the set of hypomethylated CpGs (e.g. raw beta < 0.5), the distribution plot of methylated intensity values (M) are approximately exponentially distributed, while the unmethylated intensity values (U) are approximate normally distributed (Supplementary Figure S2). The set of hypermethlated CpGs (e.g. raw beta ≥ 0.5) show the reverse: methylated probe intensity values appear to be normally distributed and unmethylated probe intensity values appear to be exponentially distributed (Supplementary Figure S2).

Based on these observed signal intensity distributions we employed an exponential-normal mixture distribution to model the signal intensity values. For each sample, we split the 450K intensity data into six parts, M and U for Infinium I probes on red channel, M and U for Infinium I probes on green channel, M for Infinium II probes on green channel and U for Infinium II probes on the red channel. We separately modeled each part of the observed intensity values as *S* = *X* + *Y*. Where *X* is the true signal, which follows a mixture of an exponential distribution and a truncated (at 0) normal distribution, i.e. $p_1 \,\exp (\lambda ) + p_2 N_ + (\nu ,\tau ^2 )$, where *p*_1_ and *p*_2_ are the proportion of data points that follow exponential or normal distribution and *p*_1_ + *p*_2_ = 1. *Y* is the background intensity, which follows a truncated (at 0) normal distribution $N_ + (\mu ,\sigma ^2 )$. Thus, the background corrected intensities can be estimated as *E*(*X*|*S* = *s*). See Supplementary Methods for detailed derivation of this quantity. Compared to the poor fit provided by a model using only an exponential distribution (as in the noob method), we find that our exponential-normal mixture model provides a good fit for methylation intensity data (Supplementary Figure S3).

The background normal distribution parameters μ and σ for each color channel can be estimated based on: 1) internal negative control probes (_neg); 2) ‘out-of-band’ signal intensities from Infinium I probes (_oob); or 3) combined M and U intensity data (_est). Different from _neg and _oob, the _est will estimate background parameters separately for Infinium I and II probes. See supplementary methods for detailed description.

## RESULTS

### Performance metrics

Methylation levels at different CpG sites across the genome of a single person can vary widely from 0% to 100%, however for any given CpG site, the methylation level in blood is usually similar from person to person with small variation (14). An important consequence of this is that the Pearson's correlation coefficient calculated on the set of ≈450K CpG sites between two unrelated individuals may be almost as high as that between two duplicate samples from the same individual. For example, the average Pearson correlation coefficient for ≈450K beta values obtained directly from raw intensity data for all possible pairs of unrelated individuals was 0.9920, while the 19 duplicate pairs (same individual) had an average Pearson's correlation coefficient of 0.9958 (see also Supplementary Figure S4A1 for a typical duplicate pair and 4B1 for an unrelated independent pair). The ≈0.4% difference in effect between identical and unrelated samples limits the usefulness of raw beta value correlation as a means of evaluating preprocessing methods. Instead, we calculated mean-centered correlation: for each CpG on the array we first calculated the mean across all samples, and then subtracted this mean from the observed value of the CpG in each sample before calculating the correlation between any two arrays. Mean-centered correlations could range from −1 (perfectly negative correlation) to 1 (perfectly positive correlation), with uncorrelated samples having an expected value of 0. The averaged mean-centered correlation for all possible pairs of unrelated samples was −0.04, whereas for duplicate sample pairs it was 0.42 (Supplementary Figure S4: A2 and B2). Thus mean-centered correlation provides a much larger difference in effect for evaluating preprocessing methods. As an alternative evaluation measure, we also calculated the absolute difference in beta values between duplicate samples at each CpG site on the array, and summarize these using the average for each duplicate pair.

### Concordance between duplicates

We separately evaluated concordance between duplicates for Infinium I and II probes. As shown in Table 1, for both types of probes ENmix performed better than the alternative methods with higher correlation and smaller methylation difference between duplicate pairs. Furthermore, unlike background subtraction methods that truncate intensity values (up to 14% of CpGs in these evaluation samples), an important feature of ENmix is that it smoothly adjusts intensity values while maintaining their relative order (Supplementary Figure S5). Overall, Q5 had better performance than other background subtraction methods, and thus we further compared ENmix_oob and Q5 in each duplicate pairs: Supplementary Figure S6 showed that ENmix_oob outperformed Q5 for the vast majority of individual duplicates.

**Table 1.** Effect of various background correction methods on methylation concordance between 19 pairs of duplicate samples was evaluated using mean-centered correlation coefficient (larger is better) and average absolute methylation difference (smaller is better)

	Infinium I				Infinium II			
Method	R*	*P*^†^	MethDiff^‡^	*P*^†^	R*	*P*^†^	Methdiff^‡^	*P*^†^
raw	0.45	ref	2.18	ref	0.39	ref	3.17	ref
q5	0.49	0.08	1.95	0.03	0.42	0.12	3.10	0.25
lumi1	0.47	0.27	2.09	0.23	0.38	0.58	3.23	0.72
lumi2	0.46	0.02	2.20	0.67	0.40	0.05	3.03	3.9×10^−3^
ENmix_est	0.52	3.9×10^−3^	1.68	1.7×10^−5^	0.45	0.03	2.97	0.02
ENmix_neg	0.50	0.04	1.85	3.5×10^−3^	0.43	0.08	3.04	0.09
ENmix_oob	0.55	7.9×10^−5^	1.49	5.6×10^−8^	0.48	7.3×10^−4^	2.75	9.8×10^−6^

*Average mean-centered correlation coefficient for 19 duplicate-pairs.

^†^Based on one-sided Student's paired *T*-test against raw result.

^‡^MethDiff: Average mean absolute methylation beta value difference (%) for 19 duplicate pairs.

Comparisons of the three parameter estimation methods for background normal distribution in ENmix model suggest that _oob performed better than _neg. Although highly correlated, estimates of background levels were higher when based on out-of-band intensities than when based on internal negative controls, particularly on the red channel (Supplementary Figure S7: A1 and A2). Estimates of background using combined methylated and unmethylated intensities (_est) were highly correlated (*r* > 0.96) with out-of-band intensities (_oob) (Supplementary Figure S7: B1 and B2). The _est separately estimates background for Infinium I and II probes, with Infinium II showing consistently higher level of background on both color channel (Supplementary Figure S7: B1 and B2).

### Accuracy

**Figure 1.**
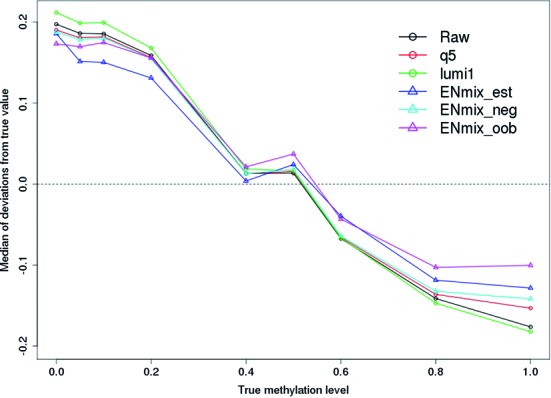
Effect of various background correction methods on methylation accuracy. Laboratory DNA methylation standards of 0 and 100% and mixtures of the two (intermediate values) were each measured on multiple arrays. Shown are medians of the CpG beta value deviation from expected methylation levels. Overall average deviation from the experimental prediction was 15.0% for raw data and 14.1% for Q5; ENmix_est, _neg and _oob had deviations of 12.3%, 13.6 and 11.6%, respectively. ENmix deviations were significantly smaller than raw, Q5 and lumi deviations (paired *T*-tests, *P* < 4 × 10^−6^).

Different levels of methylation were created by mixing unmethylated and fully methylated laboratory standard samples in varying proportions (Supplementary Figure S8). Similar to Triche *et al*. (6) we used these standards to evaluate the effect of various background correction methods on methylation measurement accuracy. Deviation from the expected methylation level was most pronounced at the two extremes of fully unmethylated or fully methylated laboratory standards: there was higher positive bias toward unmethylated states and higher negative bias toward fully methylated states (Figure 1). ENmix_est and ENmix_oob produced the most reduction in bias, followed by ENmix_neg and Q5, with lumi1 producing no reduction in bias over that observed for unadjusted data (Figure 1 and Supplementary Figure S9). Lumi2 was excluded from this analysis because the distribution of laboratory standard methylated intensity value violates the underlying model assumption resulting in substantial overcorrection (see Supplementary Figure S9).

### Probe design bias

Reflecting differences in assay chemistries, Infinium II probes produce a compressed distribution of methylation values that have different hypo- and hypermethylated modes than Infinium I probes (3,8). ENmix reduced this discrepancy so that the beta value distribution mode locations for Infinium I and II probes were closer (Supplementary Figure S10).

### Further preprocessing steps

We evaluated how ENmix performed when coupled with additional preprocessing procedures. Based on previous evaluation results (5,11,15) we selected two favorable methods: quantile normalization (5) for between-sample normalization and the beta-mixture quantile normalization (BMIQ) (11) method for correction of probe-design type bias. We applied quantile normalization separately in U and M intensity data for Infinium I and II probes. The BMIQ method was applied to methylation beta values. As shown in Supplementary Figure S11, combined use of ENmix and quantile normalization improves duplicate agreement over that of ENmix alone (paired *T*-test, *P* < 0.01) or quantile normalization alone (paired *T*-test, *P* < 10^−7^, data not shown). Similarly, BMIQ can be added to the combination of ENmix plus quantile normalization, to fully adjust probe design bias without adversely affecting agreement between duplicates (Supplementary Figure S11). Supplementary Figure S12 shows the stepwise improvements that this series of preprocessing steps produce in typical duplicate sample beta value distributions.

### Example: improvements to EWAS results

As shown in Supplementary Table S1, ENmix alone or together with other preprocessing procedures increased the number of significant CpGs identified: at FDR threshold of 0.05 ENmix alone found 12 more CpGs than were found using Q5 method. ENmix together with further preprocessing steps (ENmixDQB) found 83 more CpGs. Although we cannot assess whether the additional 83 CpGs are biologically important, we can examine the effect of ENmix correction on the *P*-values of those CpGs that have been validated in other studies of smoking. If ENmix correction improves the data, the *P*-values for validated CpGs should get smaller. Using the 26 probes previously reported by Joubert (16), we examined *P*-values in the Markunas raw data, and following ENmix or ENmix combined with other steps. Smaller *P*-values than those obtained with raw data were found for 65%, 73%, 77% and 81%, respectively for method ENmix, ENmixD, ENmixDQ, ENmixDQB. Conversely, the Q5 method decreased the statistical strength of association: Only 38% of the 26 probes had smaller *P*-values than obtained with raw data.

### Software

We implemented the proposed methods into a R package *ENmix*, that is freely available on Bioconductor web site (http://www.bioconductor.org). *ENmix* is fully compatible with several other popular R packages including *minfi* and *wateRmelon*, or can be incorporated into the *ChAMP* pipeline (see ENmix user's guide). We also developed and provided the following tools in the package to facilitate DNA methylation analysis: (i) *plotCtrl* to generate internal control plots (similar to output from Genome Studio) for data checking; (ii) *multifreqpoly* to quickly produce intuitive frequency polygon plots for data distribution inspection; (iii) *QCinfo* and *QCfilter* to extract data quality information and filter low quality samples and/or probes; (iv) *nmode.mc* to identify CpGs with multimodal beta distributions (that may result from nearby SNPs—see details in Supplementary Table S2); (v) *pcrplot* to perform principal component regression analysis and generate plots to demonstrate source of variation or to explore confounding variables for association analysis and (vi) parallel computing wrappers for methods BMIQ (11) and ComBat (17).

## DISCUSSION

Complex diseases can be associated with very small differences in DNA methylation profiles (14,18). Measurement of those profiles using Infinium HumanMethylation450 BeadChips can be affected by many experimental factors (8), which can be mitigated in part by careful data preprocessing. Background correction is the appropriate initial step in the preprocessing pipeline. But existing background correction methods have known practical or theoretical limitations, in part because the complex distribution of signal intensities is difficult to accurately model. We proposed a novel background correction method ENmix to model the methylation signal intensity with a flexible exponential-normal mixture distribution, together with a truncated normal distribution to model background noise. Evaluation results in both duplicates and experimental standard samples showed that ENmix outperformed commonly used background subtraction methods in terms of improvement in replicability and accuracy as well as reducing probe design bias. In reanalysis of previously published EWAS data, ENmix detected more CpGs and resulted in smaller *P*-values for a set of previously-validated CpGs than were obtained using raw data or Q5 background correction. ENmix is an extension of two existing model-based background correction methods: robust multi-array-average (RMA) (19) and noob (6). Like these methods ENmix assumes background intensities that are normally distributed. However, RMA and noob assume signal intensities are exponentially distributed—we demonstrate that this distribution provides poor fit to observed methylation data. In contrast, the exponential-normal distribution used by ENmix provides good fit. Furthermore, the RMA method is specifically designed for gene expression data and the background parameter estimates are problematic for some methylation data (6).

We provided three different approaches to estimate normal background distribution parameters. Similar to Triche *et al*. (6), we found that out-of-band intensity performed better than internal negative controls—perhaps reflecting improved estimates from the large set of out-of-band values versus the relatively small set of negative controls. Background estimates using the third approach in combined U and M intensities (in ENmix_est) was highly correlated with out-of-band estimates (ENmix_oob) and had similar performance. In addition ENmix_est provides separate background estimates of Infinium I and II probes, which better reduced beta value distribution differences between Infinium I and II probes. This reduction may reflect the higher Infinium II background—if unadjusted this higher background inflates both M and U intensities resulting in a compressed beta value distribution for Infinium II probes (5). We note in particular that ENmix_est has application for the analysis of publically available methylation data sets where background intensity data are not available.

After ENmix background correction the resulting data can be used with other commonly-used preprocessing methods including quantile normalization for between-sample normalization and BMIQ for further correction of probe-design bias. Together these result in stepwise complementary effects to improve data quality. We incorporated this series of preprocessing methods, along with data quality-check functions and visualization tools into *ENmix*, a multiprocessor-capable R package that facilitates large-scale analysis of methylation data.

## SUPPLEMENTARY DATA

Supplementary Data are available at NAR Online.

SUPPLEMENTARY DATA
