# Supplementary material for: ENmix: a novel background correction method for Illumina HumanMethylation450 BeadChip
Source: Nucleic Acids Res. 2015 Sep 17;44(3):e20. doi: 10.1093/nar/gkv907 (PMC4756845; doi:10.1093/nar/gkv907)
Supplement: SUPPLEMENTARY DATA [file supp_44_3_e20__index.html]

ENmix: a novel background correction method for Illumina HumanMethylation450 BeadChip — ENmix: a novel background correction method for Illumina HumanMethylation450 BeadChip — SUPPLEMENTARY DATA 

# ENmix: a novel background correction method for Illumina HumanMethylation450 BeadChip

## SUPPLEMENTARY DATA

- SUPPLEMENTARY DATA
