# Supplementary material for: ENmix: a novel background correction method for Illumina HumanMethylation450 BeadChip
Source: Nucleic Acids Res. 2015 Sep 17;44(3):e20. doi: 10.1093/nar/gkv907 (PMC4756845; doi:10.1093/nar/gkv907)
Supplement: SUPPLEMENTARY DATA [file supp_gkv907_nar-00956-met-k-2015-File003.docx]

**Title:** ENmix: a novel background correction method for Illumina HumanMethylation450 BeadChip

Zongli Xu, Liang Niu, Leping Li and Jack A. Taylor

**SUPPLEMENTARY MATERIALS**

1. **Supplementary Figures**
2. **Supplementary Tables**
3. **Supplementary Methods**
4. **Supplementary Figures**

**Supplementary Figure 1.** A) Frequency polygon plots of raw methylation beta values, B) methylated intensities and C) unmethylated intensities for Infinum II probes in the 38 samples (19 duplicates).


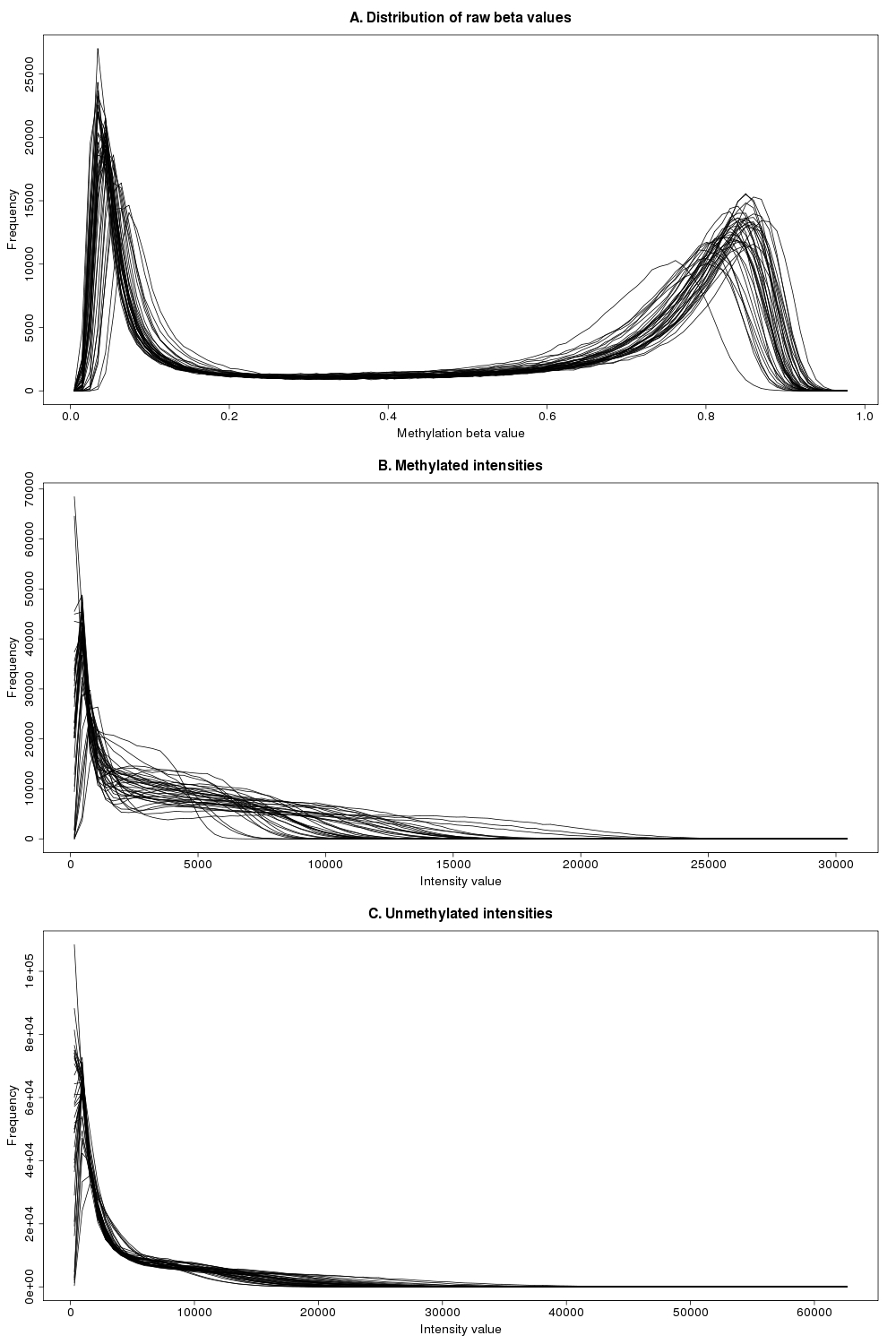


**Supplementary Figure 2**. Infinum II CpG sites were divided into two groups based on average beta value across all 38 samples (cutoff point of beta = 0.5). For CpGs with average beta < 0.5 (left two panels), the methylated intensities were approximately exponentially distributed while the unmethylated intensities follow approximately normal distributions. For the set of CpGs with average beta > 0.5 (right two panels) show the reverse.


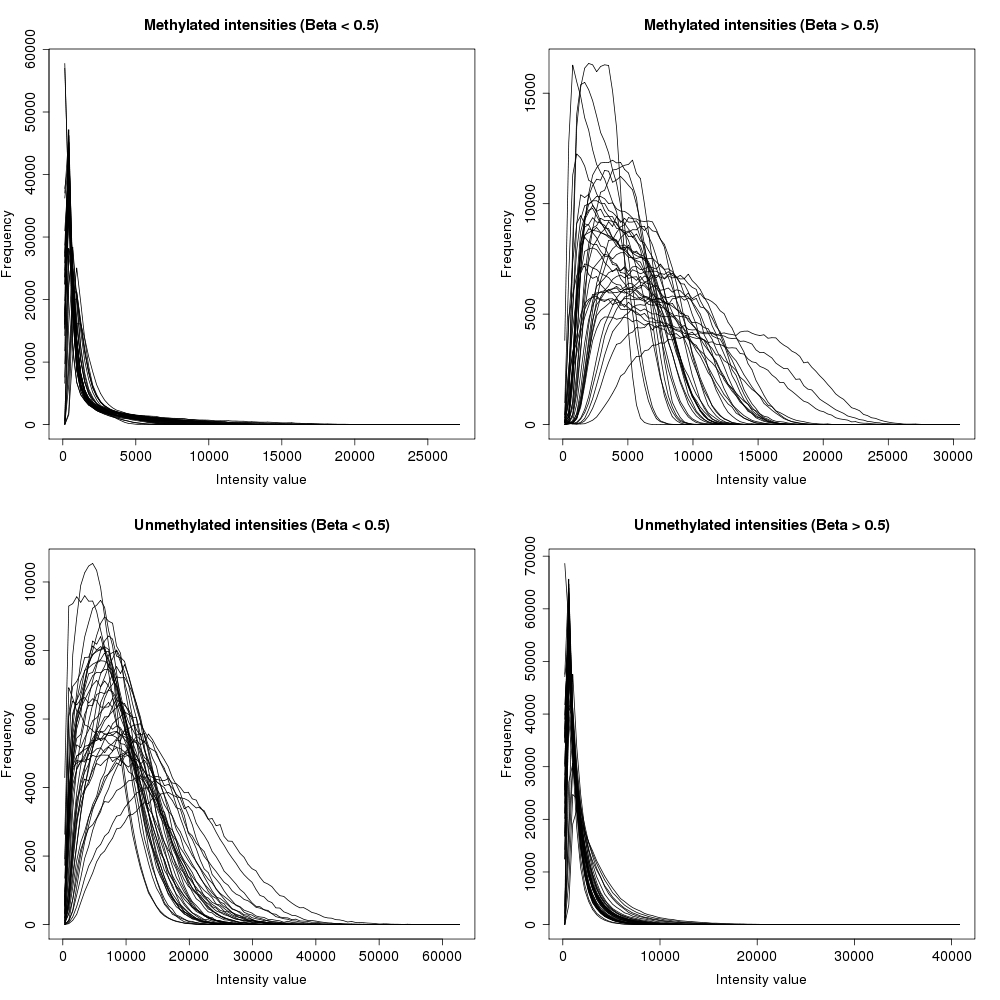


**Supplementary Figure 3**. QQ plots to show model fit of DNA methylation intensity data from Illumina Infinium HumanMethylation450 BeadChip with: A) exponential distribution for signal intensity and normal distribution for background (model assumption in Noob background correction method, model parameters were estimated using R package *minfi*); B) exponential-normal mixture distribution for signal intensity and normal distribution for background noise (model assumption in ENmix background correction method, model parameters were estimated using R package *ENmix*).


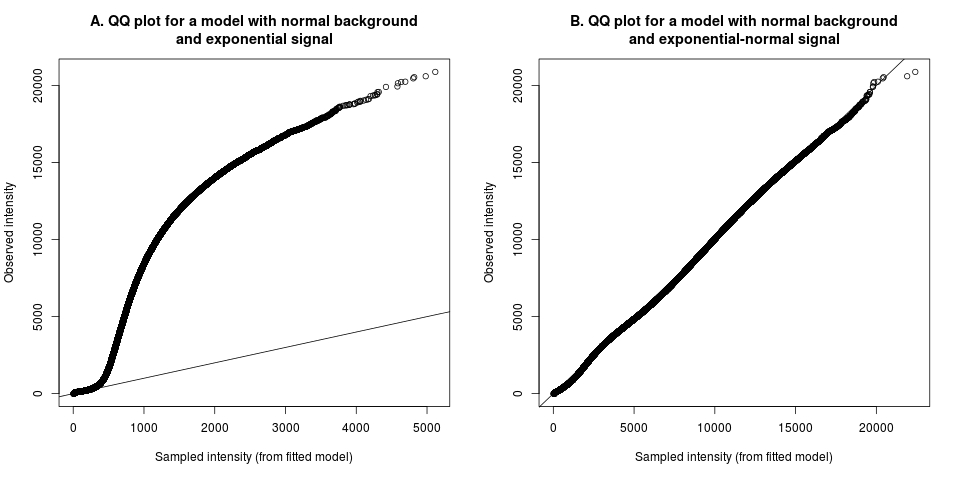


**Supplementary Figure 4**. Scatter plots of raw methylation beta values (graphs on left) and mean-centered methylation beta values (on right). A) Typical example of a duplicate pair and B) a typical pair of non-duplicate samples.


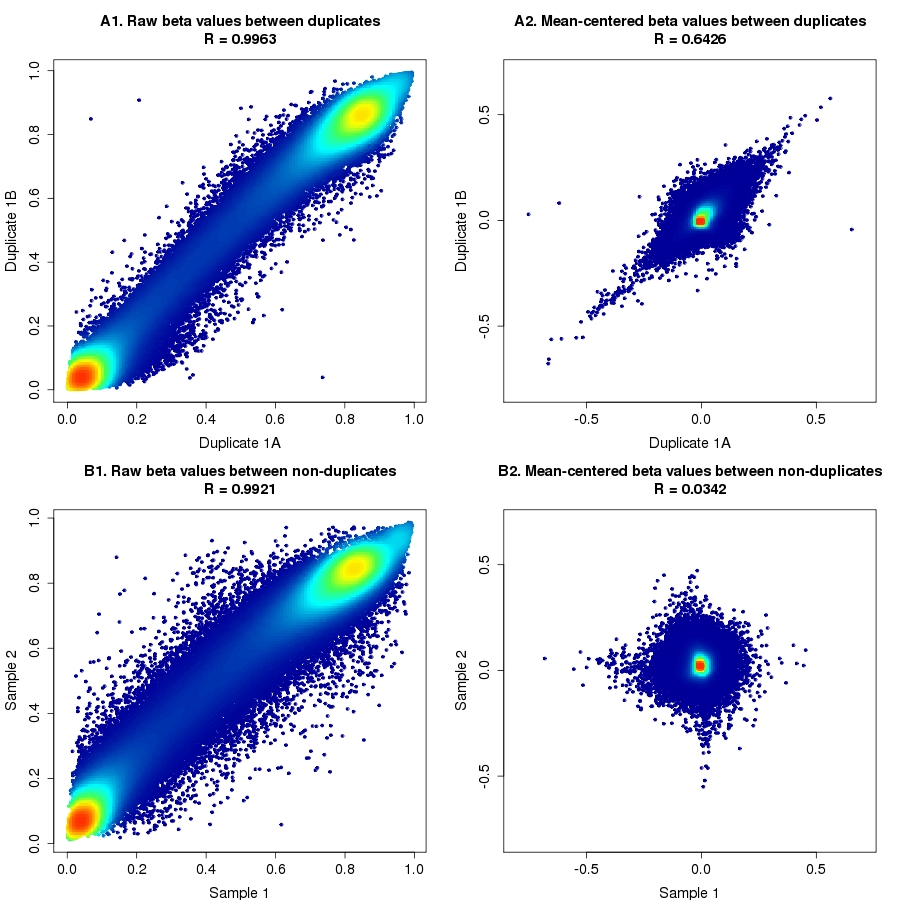


**Supplementary Figure 5.** Demonstration of the difference between ENmix and background subtraction methods on adjustment of intensity values below background estimates. Shown below is the part of raw intensity data below than background mean estimates of 640 for a typical sample in the duplicates dataset. ENmix (red circles) smoothly adjusts values downward while maintaining the monotone relationship with before adjusted values whereas background subtraction methods (Q5, lumi1 or lumi2) simply truncate these intensities (up to 14% in the 38 samples used for evaluation) or re-assign all to a single small value (in this example, 0.01).

**
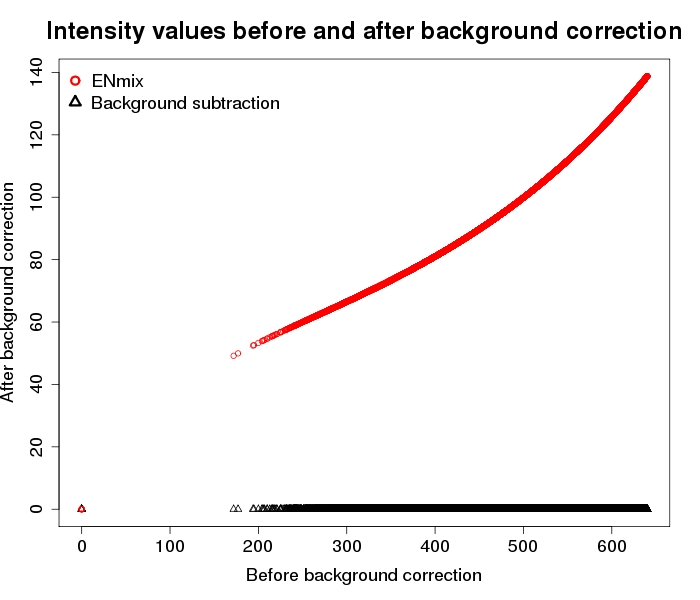
**

**Supplementary Figure 6.** Performance comparison between Q5 background subtraction method and ENmix_oob for individual duplicates. Shown are mean-centered correlation coefficients (left two graphs) and absolute differences (right two graphs) for the 19 duplicate pairs. For vast majority of duplicates, ENmix_oob performed better with higher mean-centered correlation (points falling above diagonal) and lower mean absolute difference (points falling below diagonal) for both Infinium I (top graphs) and II probes (bottom graphs).

**
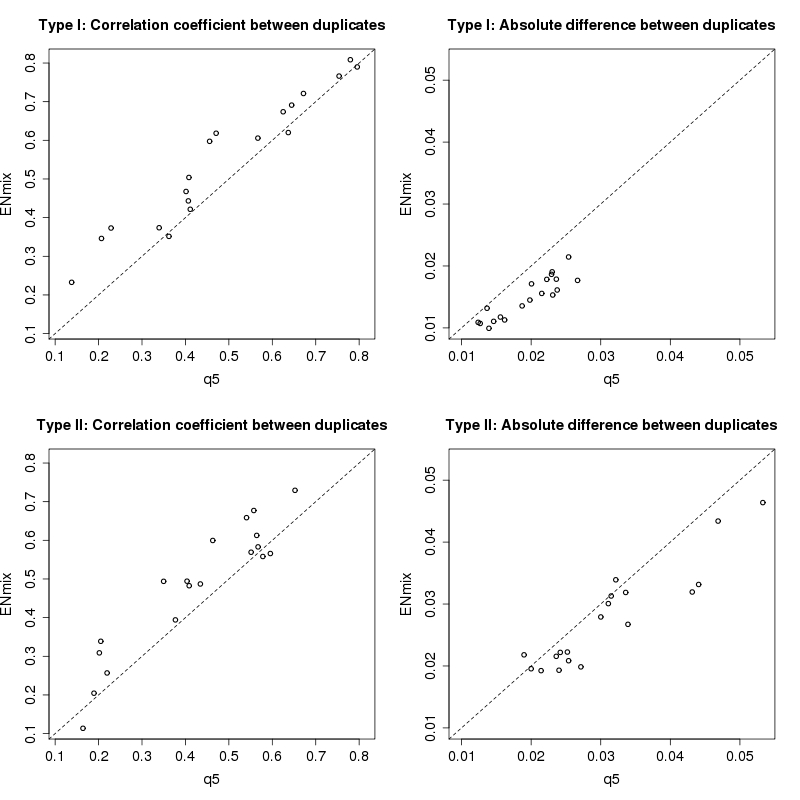
**

**Supplementary Figure 7.** Comparison of normal background mean parameter estimates for the 38 samples by different approaches: internal negative controls (_neg), Infinium I out-of-band (OOB) intensities (_oob) and combined methylated and unmethylated intensities (_est).

**
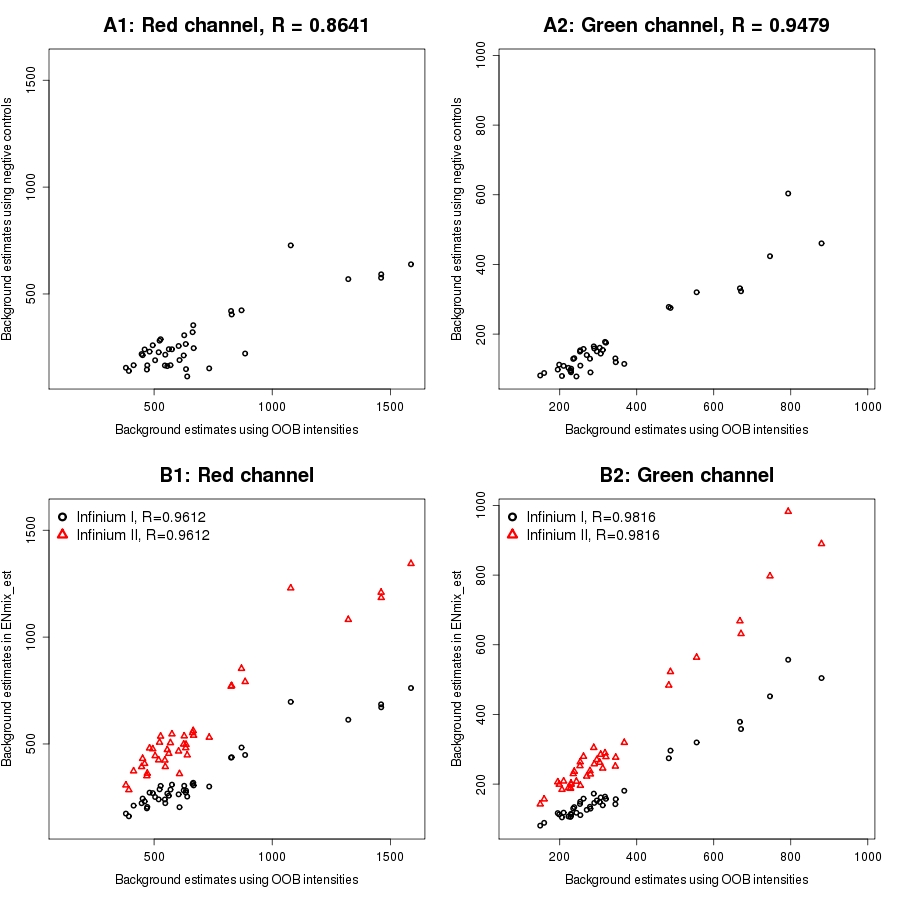
**

**Supplementary Figure 8.** Frequency polygon plots of raw methylation beta values for laboratory standard samples. 0 and 100% methylation laboratory standards were mixed together in different proportions to create samples with intermediate methylation levels. Each standard was measured independently from 2 to 10 times. Top panel: Infinium I probes. Bottom panel: Infinium II probes.

**
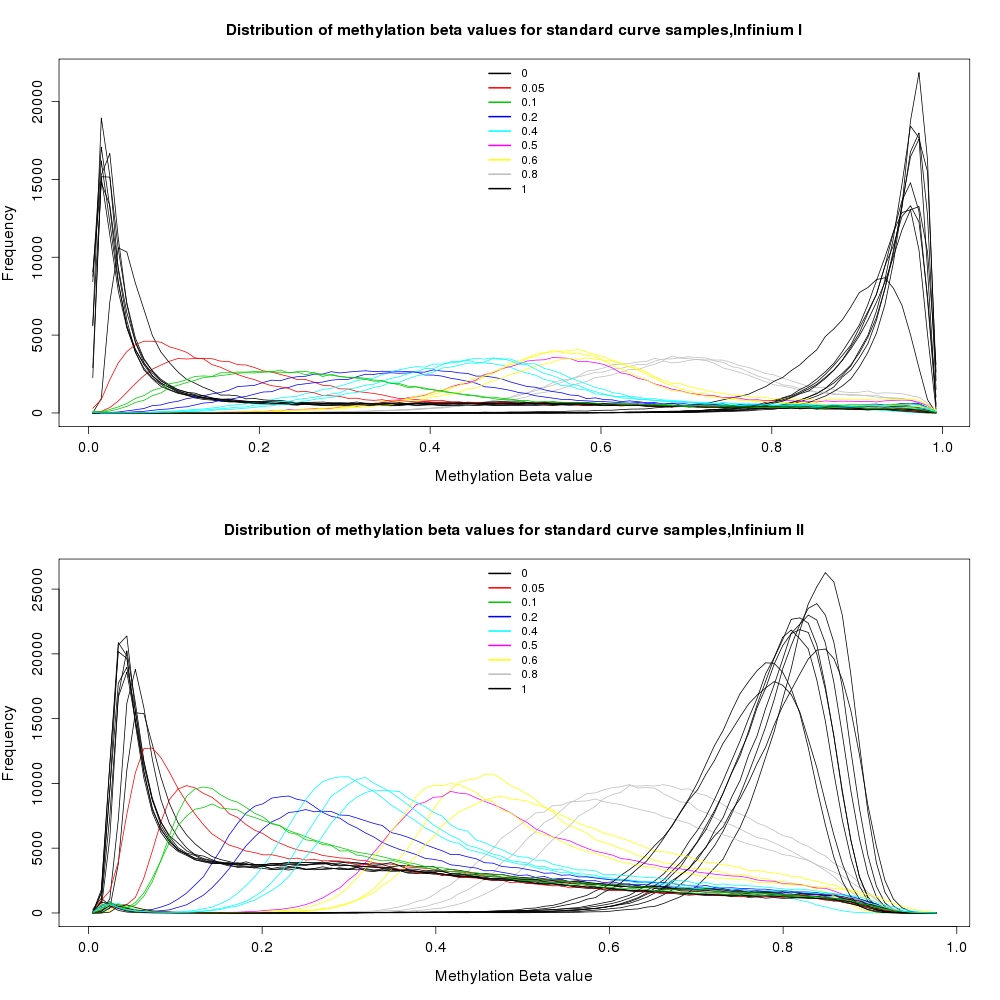
**

**Supplementary Figure 9.** Median and inter-quartile range (IQR) of methylation beta values of laboratory standards follow adjustment by different background correction methods. Dashed lines indicate expected methylation level of laboratory standard. The distribution of methylated intensity values in these samples violated the underlying assumption of method lumi2 and result in overcorrection. The results for inter-array quantile normalization and BMIQ probe type bias adjustment were added to the figure to demonstrate that these are not appropriate methods for laboratory standards due to violation of method underlying assumption. Quantile normalization assumed homogeneous samples with identical data distribution. BMIQ assumed mixed beta distribution of methylation values. Both methods can lead to inaccurate adjustment, and therefore the combined effects with background correction methods were not evaluated in these samples. **
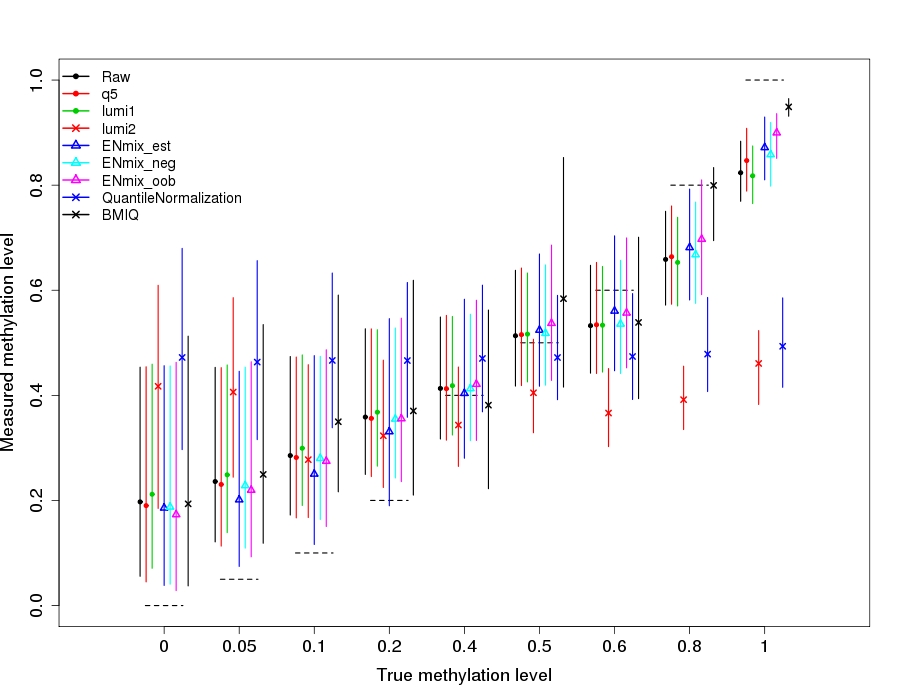
**

One-sided statistical test results using Wilcox signed rank test or pair T test.

|  | Methylation deviations (%) from TRUE | |  | Test P values against Raw | |  | Test P values against q5 | |
| --- | --- | --- | --- | --- | --- | --- | --- | --- |
|  | mean | median |  | Wilcox | T test |  | Wilcox | T test |
| raw | 14.99 | 17.70 |  | ref | ref |  | - | - |
| q5 | 14.07 | 15.67 |  | 3.84×10^-9^ | 1.95×10^-6^ |  | ref | ref |
| lumi1 | 15.79 | 17.74 |  | 0.99 | 0.99 |  | - | - |
| lumi2 | 33.94 | 29.89 |  | 1 | 1 |  | - | - |
| ENmix_est | 12.34 | 12.86 |  | 3.84×10^-9^ | 2.85×10^-9^ |  | 7.45×10^-8^ | 3.92×10^-8^ |
| ENmix_neg | 13.62 | 15.22 |  | 8.15×10^-9^ | 1.68×10^-6^ |  | 5.20×10^-8^ | 3.81×10^-6^ |
| ENmix_oob | 11.62 | 11.68 |  | 6.24×10^-8^ | 2.15×10^-7^ |  | 2.37×10^-7^ | 3.68×10^-7^ |

**Supplementary Figure 10**. Probe-type bias reduction with ENmix_oob. Shown are the absolute mode location difference between Infinium I and II methylation distributions for raw data (open circles) and ENmix_oob corrected methylation beta values (filled triangle). First (hypomethylated) modes are represented in the left panel, and second (hypermethylated) modes in the right panel. Lower ENmix values for all samples at both modes indicate that ENmix reduces probe-type bias.

**
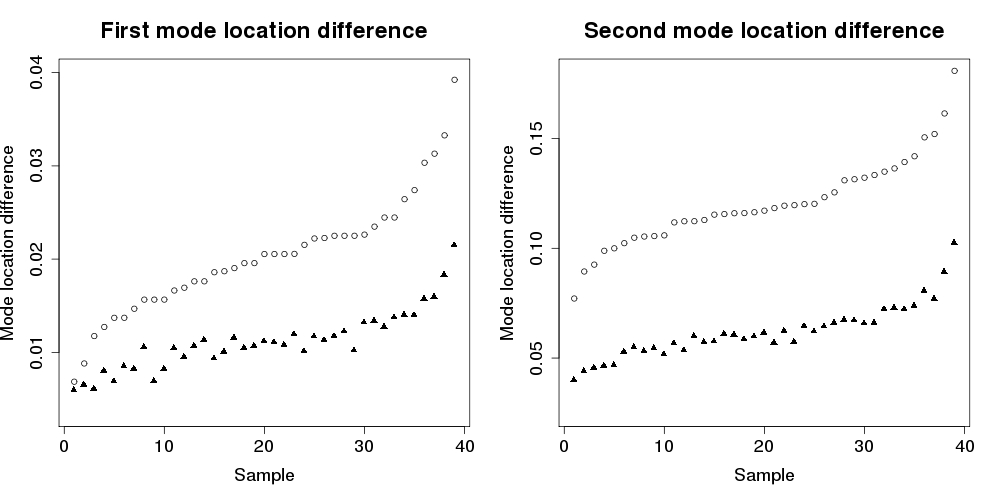
**

**Supplementary Figure 11**. Effect on concordance between duplicate samples by further preprocessing steps in conjunction with ENmix: ENmix + Quantile normalization (ENmixQ) and ENmix + Quantile normalization + BMIQ (ENmixQB). A) Mean-centered correlation B) absolute methylation difference for 19 duplicated sample pairs. Infinium I (black) and II (red) probes were plotted separately.


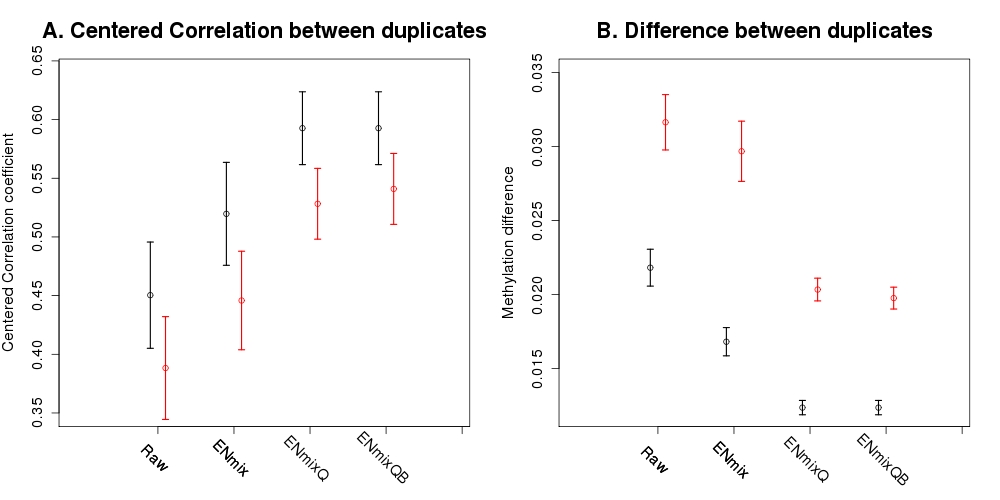


**Supplementary Figure 12**. Example of methylation beta value distributions (frequency polygon plot) for one typical duplicate pair following sequential data preprocessing steps.


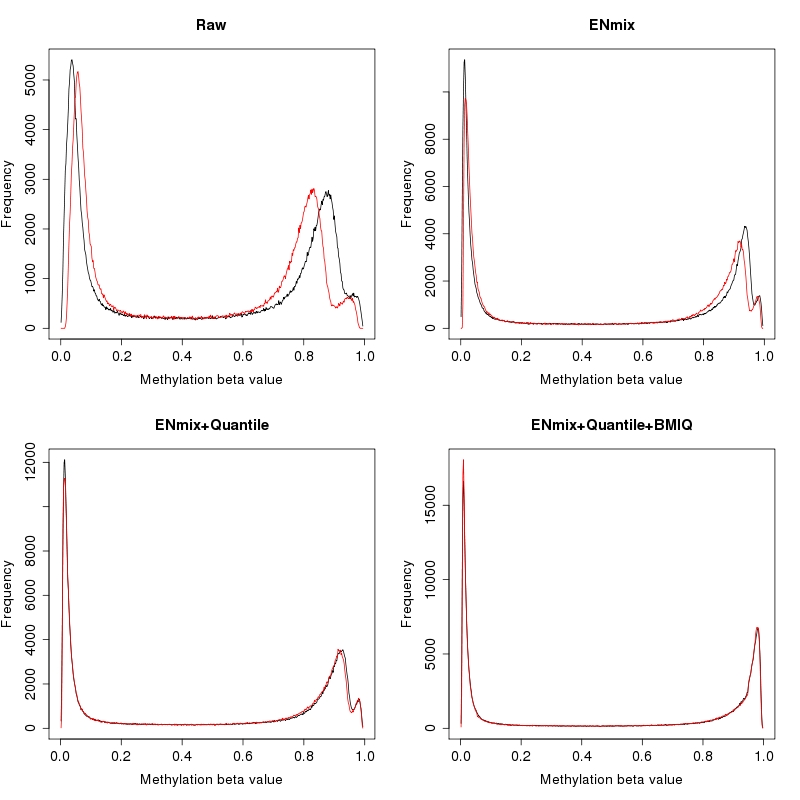


1. **Supplementary Table**

**Supplementary Table 1**. Re-analysis results of an epigenome-wide association study (EWAS) dataset (Markunas et al, EHP, 2014) in relation to maternal smoking. Blood DNA methylation data from 889 infants were preprocessed with different methods or combinations of methods: raw data, Q5 background correction, ENmix_oob background correction, ENmix and dye bias correction (ENmixD), ENmix+dye bias correction+quantile normalization (ENmixDQ), ENmix+dye bias correction + quantile normalization + BMIQ (ENmixDQB). Number of significant CpGs at q value threshold of 0.05 are summarized in column 2. Column 3: for a set of previously-validated CpGs, ENmix results in smaller p values than results obtained using raw data or q5 background correction.

| **Method** | **# of CpG with q<0.05** | **For the 26 CpGs reported by Joubert et.al, EHP 2012, the number of CpGs with smaller P value than raw data** |
| --- | --- | --- |
|  |  |  |
| Raw | 79 | Reference |
| q5 | 79 | 10 (38%) |
| ENmix | 91 | 17 (65%) |
| ENmixD | 122 | 19 (73%) |
| ENmixDQ | 156 | 20 (77%) |
| ENmixDQB | 162 | 21 (81%) |

**Supplementary Table 2**

**An empirical approach to identify multimodal distributed CpGs.** When measured in a population of people the majority of CpGs on the Illumina HumanMethylation450 BeadChip have unimodal distributions of DNA methylation values with relatively small between-person variation. However, some CpGs (typically ~10,000) may have multimodal distributions of methylation values with sizeable differences between modes and greater between-person variation. These multimodal distributed data are usually caused by SNP effect, problematic probe design, or some other unknown artifacts instead of actual methylation level and thus should be excluded from DNA methylation analysis. Although researchers have often excluded CpGs based on SNP annotation information, we found that this approach alone may exclude many well-distributed (unimodal) CpGs, while still failing to identify other multi-modal CpGs. We developed an empirical approach to identify CpGs that are not uni-modally distributed, so that researchers can make more informed decisions about whether to exclude them in their particular study populations and analyses. We have applied the approach in two different datasets: Sister study data (SS, n=200, Harlid et al, Plos one, 2015) and Norway Facial Clefts Study (NCL, n=891, Markunas et al. Environ Health Perspect. 2014) datasets. To simplify our illustration, we excluded 52,238 CpGs on X and Y chromosomes, and non-specific probes annotated by Price et al (Price et al, Epigenetics Chromatin, 2013).

From the following summary table we can see this approach (*nmode.mc* available within R package *ENmix*) can correctly identify all 65 known “SNP probes” as not unimodal in both datasets. More than 90% of CpGs with an annotated SNP in probe region are actually unimodal in both datasets. For CpGs with any SNP at CpG target site, more than 80% are unimodal, and even 50% of CpGs with an annotated common (minor allele frequency > 0.05) SNP at target site have unimodal distributions.

|  | Unimodal in NCL | | | | | | | | Concordance | % unimodal | |
| --- | --- | --- | --- | --- | --- | --- | --- | --- | --- | --- | --- |
|  | Yes | | |  | | No | | |  |  |  |
|  | Unimodal in SS | | | |  | Unimodal in SS | | |  | NCL | SS |
|  | yes |  | no | |  | yes |  | No |  |  |  |
| SNP probe | 0 |  | 0 | |  | 0 |  | 65 | 1.00 | 0 | 0 |
| SNP in target site  with MAF ≥0.05* | 2785 |  | 493 | |  | 315 |  | 2584 | 0.87 | 0.531 | 0.502 |
| SNP in target site^#^ | 14131 |  | 810 | |  | 409 |  | 2844 | 0.93 | 0.821 | 0.799 |
| SNP in probe ^#^ | 109480 |  | 2140 | |  | 676 |  | 3227 | 0.98 | 0.966 | 0.954 |
| No SNP in probe^#^ | 314502 |  | 2264 | |  | 443 |  | 542 | 0.99 | 0.997 | 0.991 |

*Based on 1000 genome project data for European population (http://www.1000genomes.org/)

# based on annotation by Price et al, Epigenetics Chromatin, 2013

1. **Supplementary Methods**

**Derivation of exponential-normal mixture signal intensity background correction model**

Let $S$ be the observed intensity. We assume that $S$ is the sum of a signal component $X$ and a background noise component $Y$, i.e., $S=X+Y$, with $X$ and $Y$ independent. Since the histogram of $S$ is usually bimodal, we assume that $X$ follows a mixture of an exponential distribution and a truncated (at 0) normal distribution, i.e., $p_{1}\exp\left( \lambda\right)+p_{2}N_{+}( \nu,\tau^{2})$ and that $Y$ follows a truncated (at 0) normal distribution $N_{+}( \mu,\sigma^{2})$. Notice that the above two normal distributions are truncated at 0, since both $X$ and $Y$ are positive. Under this model, we define the background corrected intensity for an observed density $s$ as $E(X|S=s)$. A formula for this quantity is derived below.

Let $\boldsymbol{\Phi}\left( z \right)$ and $\phi\left( z \right)$ be the standard normal cumulative distribution function and density function respectively. Specifically

$$\boldsymbol{\Phi}\left( z \right)=\int_{-\infty}^{z} \frac{1}{\sqrt{2\pi}}\exp\left( -\frac{1}{2}\omega^{2} \right)d\omega$$

and

$$\phi\left( z \right)=\frac{1}{\sqrt{2\pi}}\exp\left( -\frac{1}{2}z^{2} \right).$$

Then the density of the joint distribution of $X$ and $Y$ is given by

$$f_{X,Y}\left( x,y \right)=\left( p_{1}\lambda\exp\left( -\lambda x \right)+p_{2}C_{2}\frac{1}{\tau}\phi\left( \frac{x-\nu}{\tau} \right) \right)\cdot C\frac{1}{\sigma}\phi\left( \frac{y-\mu}{\sigma} \right),$$

where $x>0$, $y>0$ and $C=1/(1-\boldsymbol{\Phi}\left( -\frac{\mu}{\sigma} \right))=1/\boldsymbol{\Phi}\left( \frac{\mu}{\sigma} \right)$ and $C_{2}=1/\left( 1-\boldsymbol{\Phi}\left( -\frac{\nu}{\tau} \right) \right)=1/\boldsymbol{\Phi}\left( \frac{\nu}{\tau} \right)$. Therefore, the joint distribution of $X$ and $S$ is

$$f_{X,S}\left( x,s \right)=f_{X,Y}\left( x,s-x \right)|J|$$

where $J$ is the Jacobian of the transformation. Notice that $J=1$, thus

$$f_{X,S}\left( x,s \right)=\left( p_{1}\lambda\exp\left( -\lambda x \right)+p_{2}C_{2}\frac{1}{\tau}\phi\left( \frac{x-\nu}{\tau} \right) \right)\cdot C\frac{1}{\sigma}\phi\left( \frac{s-x-\mu}{\sigma} \right).$$

The conditional expectation of $X$ given $S=s$ is

$$E\left( x | S=s \right)=\int_{0}^{s} {x\cdot f}_{X|S}\left( x|s \right)dx=\frac{\int_{0}^{s} {x\cdot f}_{X,S}\left( x,s \right)dx}{\int_{0}^{s} f_{X,S}\left( x,s \right)dx}$$

where the denominator (the marginal density function of $s$) is

$$\int_{0}^{s} \left( p_{1}\lambda\exp\left( -\lambda x \right)+p_{2}C_{2}\frac{1}{\tau}\phi\left( \frac{x-\nu}{\tau} \right) \right)\cdot C\frac{1}{\sigma}\phi\left( \frac{s-x-\mu}{\sigma} \right)dx$$

$$=\int_{0}^{s} p_{1}\lambda\exp\left( -\lambda x \right)\cdot C\frac{1}{\sigma}\phi\left( \frac{x-s+\mu}{\sigma} \right)dx+\int_{0}^{s} p_{2}C_{2}\frac{1}{\tau}\phi\left( \frac{x-\nu}{\tau} \right)\cdot C\frac{1}{\sigma}\phi\left( \frac{s-x-\mu}{\sigma} \right)dx.$$

Now we consider the two integrals on the right hand side separately. For $\int_{0}^{s} p_{1}\lambda\exp\left( -\lambda x \right)\cdot C\frac{1}{\sigma}\phi\left( \frac{x-s+\mu}{\sigma} \right)dx$, let $\omega=\frac{x-s+\mu}{\sigma}$ so that $d\omega=\frac{1}{\sigma}dx$, then

$$\int_{0}^{s} p_{1}\lambda\exp\left( -\lambda x \right)\cdot C\frac{1}{\sigma}\phi\left( \frac{x-s+\mu}{\sigma} \right)dx$$

$$=p_{1}\lambda C\int_{\frac{-s+\mu}{\sigma}}^{\frac{\mu}{\sigma}} \exp\left( -\lambda\left( \sigma\omega+s-\mu\right) \right)\phi\left( \omega\right)d\omega$$

$$=p_{1}\lambda C\cdot\exp\left( -\lambda\left( s-\mu\right) \right)\int_{\frac{-s+\mu}{\sigma}}^{\frac{\mu}{\sigma}} \exp\left( -\lambda\sigma\omega\right)\frac{1}{\sqrt{2\pi}}\exp\left( -\frac{1}{2}\omega^{2} \right)d\omega$$

$$=p_{1}\lambda C\cdot\exp\left( -\lambda\left( s-\mu\right) \right)\exp\left( \frac{1}{2}\lambda^{2}\sigma^{2} \right)\int_{\frac{-s+\mu}{\sigma}}^{\frac{\mu}{\sigma}} \frac{1}{\sqrt{2\pi}}\exp\left( -\frac{1}{2}\left( \omega+\lambda\sigma\right)^{2} \right)d\omega$$

$$=p_{1}\lambda C\cdot\exp\left( \frac{1}{2}\lambda^{2}\sigma^{2}-\lambda\left( s-\mu\right) \right)\left( \boldsymbol{\Phi}\left( \frac{\mu+\lambda\sigma^{2}}{\sigma} \right)-\boldsymbol{\Phi}\left( \frac{-s+\mu+\lambda\sigma^{2}}{\sigma} \right) \right)$$

$$=p_{1}\lambda C\cdot\exp\left( \frac{1}{2}\lambda^{2}\sigma^{2}-\lambda\left( s-\mu\right) \right)\left( \boldsymbol{\Phi}\left( \frac{a}{\sigma} \right)-\boldsymbol{\Phi}\left( \frac{-s+a}{\sigma} \right) \right),$$

where $a=\mu+\lambda\sigma^{2}$. For $\int_{0}^{s} p_{2}C_{2}\frac{1}{\tau}\phi\left( \frac{x-\nu}{\tau} \right)\cdot C\frac{1}{\sigma}\phi\left( \frac{s-x-\mu}{\sigma} \right)dx$, we have

$$\int_{0}^{s} p_{2}C_{2}\frac{1}{\tau}\phi\left( \frac{x-\nu}{\tau} \right)\cdot C\frac{1}{\sigma}\phi\left( \frac{s-x-\mu}{\sigma} \right)dx$$

$$=p_{2}C_{2}C\int_{0}^{s} \frac{1}{\sqrt{2\pi}\tau}\exp\left( -\frac{\left( x-\nu\right)^{2}}{2\tau^{2}} \right)\cdot\frac{1}{\sqrt{2\pi}\sigma}\exp\left( -\frac{\left( s-x-\mu\right)^{2}}{2\sigma^{2}} \right)dx$$

$$=p_{2}C_{2}C\frac{1}{\sqrt{2\pi\left( \tau^{2}+\sigma^{2} \right)}}\exp\left( -\frac{\left( s-\left( \nu+\mu\right) \right)^{2}}{2\left( \tau^{2}+\sigma^{2} \right)} \right)\int_{0}^{s} \frac{1}{\sqrt{2\pi}\frac{\tau\sigma}{\sqrt{\tau^{2}+\sigma^{2}}}}\exp\left( -\frac{\left( x-\frac{\tau^{2}\left( s-\mu\right)+\sigma^{2}\nu}{\tau^{2}+\sigma^{2}} \right)^{2}}{2\left( \frac{\tau\sigma}{\sqrt{\tau^{2}+\sigma^{2}}} \right)^{2}} \right)dx$$

$$=p_{2}C_{2}C\frac{1}{\sqrt{2\pi\left( \tau^{2}+\sigma^{2} \right)}}\exp\left( -\frac{\left( s-\left( \nu+\mu\right) \right)^{2}}{2\left( \tau^{2}+\sigma^{2} \right)} \right)\left( \boldsymbol{\Phi}\left( \frac{s-A}{B} \right)-\boldsymbol{\Phi}\left( -\frac{A}{B} \right) \right),$$

where $A=\frac{\tau^{2}\left( s-\mu\right)+\sigma^{2}\nu}{\tau^{2}+\sigma^{2}}$ and $B=\frac{\tau\sigma}{\sqrt{\tau^{2}+\sigma^{2}}}$. Therefore the denominator is

$$p_{1}\lambda C\exp\left( \frac{1}{2}\lambda^{2}\sigma^{2}-\lambda\left( s-\mu\right) \right)\left( \boldsymbol{\Phi}\left( \frac{a}{\sigma} \right)-\boldsymbol{\Phi}\left( \frac{-s+a}{\sigma} \right) \right)+p_{2}C_{2}C\frac{1}{\sqrt{2\pi\left( \tau^{2}+\sigma^{2} \right)}}\exp\left( -\frac{\left( s-\left( \nu+\mu\right) \right)^{2}}{2\left( \tau^{2}+\sigma^{2} \right)} \right)\left( \boldsymbol{\Phi}\left( \frac{s-A}{B} \right)-\boldsymbol{\Phi}\left( -\frac{A}{B} \right) \right).$$

The numerator is

$$\int_{0}^{s} {x\cdot f}_{X,S}\left( x,s \right)dx$$

$$=\int_{0}^{s} xp_{1}\lambda\exp\left( -\lambda x \right)\cdot C\frac{1}{\sigma}\phi\left( \frac{x-s+\mu}{\sigma} \right)dx+\int_{0}^{s} xp_{2}C_{2}\frac{1}{\tau}\phi\left( \frac{x-\nu}{\tau} \right)\cdot C\frac{1}{\sigma}\phi\left( \frac{s-x-\mu}{\sigma} \right)dx.$$

Again we consider the two integrals on the right hand side separately. For $\int_{0}^{s} xp_{1}\lambda\exp\left( -\lambda x \right)\cdot C\frac{1}{\sigma}\phi\left( \frac{x-s+\mu}{\sigma} \right)dx$, let $\omega=\frac{x-s+\mu}{\sigma}$ so that $d\omega=\frac{1}{\sigma}dx$, then

$$\int_{0}^{s} xp_{1}\lambda\exp\left( -\lambda x \right)\cdot C\frac{1}{\sigma}\phi\left( \frac{x-s+\mu}{\sigma} \right)dx$$

$$=p_{1}\lambda C\cdot\exp\left( \frac{1}{2}\lambda^{2}\sigma^{2}-\lambda\left( s-\mu\right) \right)\int_{\frac{-s+\mu}{\sigma}}^{\frac{\mu}{\sigma}} \left( \sigma\omega+s-\mu\right)\frac{1}{\sqrt{2\pi}}\exp\left( -\frac{1}{2}\left( \omega+\lambda\sigma\right)^{2} \right)d\omega,$$

Let $z=\omega+\lambda\sigma,$ the above integral becomes

$$p_{1}\lambda C\cdot\exp\left( \frac{1}{2}\lambda^{2}\sigma^{2}-\lambda\left( s-\mu\right) \right)\int_{\frac{-s+\mu+\lambda\sigma^{2}}{\sigma}}^{\frac{\mu+\lambda\sigma^{2}}{\sigma}} \left( \sigma z-\lambda\sigma^{2}+s-\mu\right)\frac{1}{\sqrt{2\pi}}\exp\left( -\frac{1}{2}z^{2} \right)dz$$

$$=p_{1}\lambda C\cdot\exp\left( \frac{1}{2}\lambda^{2}\sigma^{2}-\lambda\left( s-\mu\right) \right)\left( \sigma\left( \phi\left( \frac{-s+\mu+\lambda\sigma^{2}}{\sigma} \right)-\phi\left( \frac{\mu+\lambda\sigma^{2}}{\sigma} \right) \right)+\left( s-\mu-\lambda\sigma^{2} \right)\cdot\left( \boldsymbol{\Phi}\left( \frac{\mu+\lambda\sigma^{2}}{\sigma} \right)-\boldsymbol{\Phi}\left( \frac{-s+\mu+\lambda\sigma^{2}}{\sigma} \right) \right) \right)$$

$$=p_{1}\lambda C\cdot\exp\left( \frac{1}{2}\lambda^{2}\sigma^{2}-\lambda\left( s-\mu\right) \right)\left( \sigma\left( \phi\left( \frac{-s+a}{\sigma} \right)-\phi\left( \frac{a}{\sigma} \right) \right)+\left( s-a \right)\cdot\left( \boldsymbol{\Phi}\left( \frac{a}{\sigma} \right)-\boldsymbol{\Phi}\left( \frac{-s+a}{\sigma} \right) \right) \right).$$

For $\int_{0}^{s} xp_{2}C_{2}\frac{1}{\tau}\phi\left( \frac{x-\nu}{\tau} \right)\cdot C\frac{1}{\sigma}\phi\left( \frac{s-x-\mu}{\sigma} \right)dx$, we have

$$\int_{0}^{s} xp_{2}C_{2}\frac{1}{\tau}\phi\left( \frac{x-\nu}{\tau} \right)\cdot C\frac{1}{\sigma}\phi\left( \frac{s-x-\mu}{\sigma} \right)dx$$

$$=p_{2}C_{2}C\frac{1}{\sqrt{2\pi\left( \tau^{2}+\sigma^{2} \right)}}\exp\left( -\frac{\left( s-\left( \nu+\mu\right) \right)^{2}}{2\left( \tau^{2}+\sigma^{2} \right)} \right)\int_{0}^{S} \frac{x}{\sqrt{2\pi}B}\exp\left( -\frac{\left( x-A \right)^{2}}{2B^{2}} \right)dx.$$

Let $t=\frac{\left( x-A \right)^{2}}{2B^{2}}$, then $\left( x-A \right)dx=B^{2}dt$ and the integral on the right hand side becomes

$$\int_{0}^{S} \frac{x-A+A}{\sqrt{2\pi}B}\exp\left( -\frac{\left( x-A \right)^{2}}{2B^{2}} \right)dx$$

$$=\int_{\frac{A^{2}}{2B^{2}}}^{\frac{\left( s-A \right)^{2}}{2B^{2}}} \frac{B^{2}}{\sqrt{2\pi}B}\exp\left( -t \right)dt+\int_{0}^{S} \frac{A}{\sqrt{2\pi}B}\exp\left( -\frac{\left( x-A \right)^{2}}{2B^{2}} \right)dx$$

$$=\frac{B}{\sqrt{2\pi}}\left( \exp\left( -\frac{A^{2}}{2B^{2}} \right)-\exp\left( -\frac{\left( s-A \right)^{2}}{2B^{2}} \right) \right)+A(\boldsymbol{\Phi}\left( \frac{s-A}{B} \right)-\boldsymbol{\Phi}(-\frac{A}{B}))$$

Therefore

$$\int_{0}^{s} xp_{2}C_{2}\frac{1}{\tau}\phi\left( \frac{x-\nu}{\tau} \right)\cdot C\frac{1}{\sigma}\phi\left( \frac{s-x-\mu}{\sigma} \right)dx$$

$$=p_{2}C_{2}C\frac{1}{\sqrt{2\pi\left( \tau^{2}+\sigma^{2} \right)}}\exp\left( -\frac{\left( s-\left( \nu+\mu\right) \right)^{2}}{2\left( \tau^{2}+\sigma^{2} \right)} \right)\left( \frac{B}{\sqrt{2\pi}}\left( \exp\left( -\frac{A^{2}}{2B^{2}} \right)-\exp\left( -\frac{\left( s-A \right)^{2}}{2B^{2}} \right) \right)+A\left( \boldsymbol{\Phi}\left( \frac{s-A}{B} \right)-\boldsymbol{\Phi}\left( -\frac{A}{B} \right) \right) \right).$$

So the numerator

$$\int_{0}^{s} {x\cdot f}_{X,S}\left( x,s \right)dx$$

$$=p_{1}\lambda C\cdot\exp\left( \frac{1}{2}\lambda^{2}\sigma^{2}-\lambda\left( s-\mu\right) \right)\left( \sigma\left( \phi\left( \frac{-s+a}{\sigma} \right)-\phi\left( \frac{a}{\sigma} \right) \right)+\left( s-a \right)\cdot\left( \boldsymbol{\Phi}\left( \frac{a}{\sigma} \right)-\boldsymbol{\Phi}\left( \frac{-s+a}{\sigma} \right) \right) \right)+p_{2}C_{2}C\frac{1}{\sqrt{2\pi\left( \tau^{2}+\sigma^{2} \right)}}\exp\left( -\frac{\left( s-\left( \nu+\mu\right) \right)^{2}}{2\left( \tau^{2}+\sigma^{2} \right)} \right)\left( \frac{B}{\sqrt{2\pi}}\left( \exp\left( -\frac{A^{2}}{2B^{2}} \right)-\exp\left( -\frac{\left( s-A \right)^{2}}{2B^{2}} \right) \right)+A\left( \boldsymbol{\Phi}\left( \frac{s-A}{B} \right)-\boldsymbol{\Phi}\left( -\frac{A}{B} \right) \right) \right)$$

We use an ad-hoc approach to estimate the parameters $\mu,\sigma,\lambda, \nu, \tau, p_{1}$ and $p_{2}$. The background normal distribution parameters $\mu$ and $\sigma$ for each color channel can be estimated from: 1) internal negative control probes (_neg) , 2) “out-of-band” signal intensities from Infinium I probes (_oob); or 3) combined M and U intensity data (_est). We used a robust estimation method proposed by Huber PJ (Huber P.J. The Annals of mathematical statistics, 1963) to estimate mean parameter $\mu$ and variance $\sigma$ from internal negative controls or out-of-band intensities. We observed that intensity values for Infinium II were often higher than Infinium I and thus the corresponding backgrounds were estimated separately in _est: for Infinium I probes, we combined M and U intensities at each color channel and estimated the mode positions $\hat{\mu}_{Ired}$ and $\hat{\mu}_{Igrn}$ using kernel density function; For Infinium II probes, we first estimated mode position $\hat{\mu}_{II}$ in U and M combined intensity data, and then modified Infinium I estimates based on relative magnitude to get corresponding background estimates on red and green channel.

The corresponding variation parameter $\sigma$ was estimated as $\hat{\sigma}=\sqrt{\frac{\sum_{s_{i}<\hat{\mu}} {(s_{i}-\hat{\mu})}^{2}}{m}}$ for each type of probes at red or green channel, where $m$ is the number of observed intensities that were less than $\hat{\mu}$.

To estimate the other parameters $\lambda, \nu, \tau, p_{1}$ and $p_{2}$, we make use of the above estimates $\hat{\mu}$ and $\hat{\sigma}$. Specifically, we exclude the $m$ observed intensities that are less than $\hat{\mu}$ , subtract $\hat{\mu}$ from each of the remaining $n-m$ observed intensities and fit a mixture distribution $p_{1}^{'}\exp\left( \lambda^{'} \right)+p_{2}^{'}N(\nu^{'},{\tau^{'}}^{2})$ to the resulted data, and then calculate the maximum likelihood estimates (MLEs) of $\lambda^{'}, \nu^{'}, \tau^{'}, p_{1}^{'}$ and $p_{2}^{'}$ using an expectation-maximization (EM) algorithm. Suppose the MLEs are $\hat{\lambda^{'}}, \hat{\nu^{'}}, \hat{\tau^{'}}, \hat{p_{1}^{'}}$ and $\hat{p_{2}^{'}}$, then the $\lambda, \nu, \tau, p_{1}$ and $p_{2}$ are estimated as

$$\hat{\lambda}=\hat{\lambda^{'}}$$

$$\hat{\nu}=\hat{\nu^{'}}$$

$$\hat{\tau}=\sqrt{{\hat{\tau^{'}}}^{2}-\hat{\sigma}^{2}}$$

$$\hat{p_{1}}=\frac{m+\left( n-m \right)\hat{p_{1}^{'}}}{n}$$

$$\hat{p_{2}}=\frac{(n-m)\hat{p_{2}^{'}}}{n}.$$

The EM algorithm to find $\hat{\lambda^{'}}, \hat{\nu^{'}}, \hat{\tau^{'}}, \hat{p_{1}^{'}}$ and $\hat{p_{2}^{'}}$ are described as follows.

The EM algorithm aims to find the MLE of $\lambda^{'}, \nu^{'}, \tau^{'}, p_{1}^{'}$ and $p_{2}^{'}$ based on observations $Z_{i}=z_{i} \mathrm{for} 1\leq i\leq n-m$, where $Z_{i}$ independently follows a distribution $p_{1}^{'}\exp\left( \lambda^{'} \right)+p_{2}^{'}N(\nu^{'},{\tau^{'}}^{2})$. The indicator variable

$$I_{i}=\left\{ \begin{aligned} 1, with probability p_{1}^{'} \\ 0, with probability p_{2}^{'} \end{aligned} \right.$$

that indicates the distribution from which $Z_{i}$ is generated (either an exponential distribution $\exp\left( \lambda^{'} \right)$ or a normal distribution $N(\nu^{'},{\tau^{'}}^{2})$), is treated as the unobserved latent variable, where $1\leq i\leq n-m$. The E step involves calculating the expected value of complete log-likelihood function with respect to the conditional distribution of $\boldsymbol{I}=(I_{1},I_{2},\cdots, I_{n-m})$ under the current estimate at iteration $t$, namely $\boldsymbol{p}^{t}=({\hat{\lambda^{'}}}^{t}, {\hat{\nu^{'}}}^{t}, {\hat{\tau^{'}}}^{t}, {\hat{p_{1}^{'}}}^{t}, {\hat{p_{2}^{'}}}^{t})$. That is, we calculate

$$E_{\boldsymbol{I}|\boldsymbol{Z},\boldsymbol{p}^{t}}\left( \log L\left( \boldsymbol{p} \right) \right)=E_{\boldsymbol{I}|\boldsymbol{Z},\boldsymbol{p}^{t}}(\log P\left( \boldsymbol{Z}, \boldsymbol{I} \right|\boldsymbol{p}))$$

$$=\sum_{i=1}^{n-m} E_{\boldsymbol{I}|\boldsymbol{Z},\boldsymbol{P}^{t}}(\log P\left( Z_{n}, I_{n} \right|\boldsymbol{P}))$$

$$=\sum_{i=1}^{n-m} \left( w_{i1}\log\left( p_{1}^{'}\lambda^{'}\exp\left( -\lambda^{'}z_{i} \right) \right)+w_{i2}\log\left( p_{2}^{'}\frac{1}{\sqrt{2\pi{\tau^{'}}^{2}}}\exp\left( -\frac{\left( z_{i}-\nu^{'} \right)^{2}}{2{\tau^{'}}^{2}} \right) \right) \right)$$

$$=\sum_{i=1}^{n-m} \left( w_{i1}( \log p_{1}^{'}+log\lambda^{'}-\lambda^{'}z_{i} \right)+w_{i2}(\log p_{2}^{'}+\log\left( \frac{1}{\sqrt{2\pi{\tau^{'}}^{2}}} \right)-\frac{\left( z_{i}-\nu^{'} \right)^{2}}{2{\tau^{'}}^{2}})),$$

where

$$w_{i1}=\frac{{\hat{p_{1}^{'}}}^{t}\cdot{\hat{\lambda^{'}}}^{t}\exp\left( -{\hat{\lambda^{'}}}^{t}z_{i} \right)}{{\hat{p_{1}^{'}}}^{t}\cdot{\hat{\lambda^{'}}}^{t}\exp\left( -{\hat{\lambda^{'}}}^{t}z_{i} \right)+{\hat{p_{2}^{'}}}^{t}\cdot\frac{1}{\sqrt{2\pi{({\hat{\tau^{'}}}^{t})}^{2}}}\exp\left( -\frac{\left( z_{i}-{\hat{\nu^{'}}}^{t} \right)^{2}}{2{({\hat{\tau^{'}}}^{t})}^{2}} \right)}$$

$$w_{i2}=\frac{{\hat{p_{2}^{'}}}^{t}\cdot\frac{1}{\sqrt{2\pi{({\hat{\tau^{'}}}^{t})}^{2}}}\exp\left( -\frac{\left( z_{i}-{\hat{\nu^{'}}}^{t} \right)^{2}}{2{({\hat{\tau^{'}}}^{t})}^{2}} \right)}{{\hat{p_{1}^{'}}}^{t}\cdot{\hat{\lambda^{'}}}^{t}\exp\left( -{\hat{\lambda^{'}}}^{t}z_{i} \right)+{\hat{p_{2}^{'}}}^{t}\cdot\frac{1}{\sqrt{2\pi{({\hat{\tau^{'}}}^{t})}^{2}}}\exp\left( -\frac{\left( z_{i}-{\hat{\nu^{'}}}^{t} \right)^{2}}{2{({\hat{\tau^{'}}}^{t})}^{2}} \right)}.$$

The M step involves maximizing $E_{\boldsymbol{I}|\boldsymbol{Z},\boldsymbol{p}^{t}}\left( \log L\left( \boldsymbol{p} \right) \right)$ under the constraint $p_{1}^{'}+p_{2}^{'}=1$ to update $\boldsymbol{p}^{t}$ to $\boldsymbol{p}^{t+1}$. This maximization uses the Lagrange multiplier technique; we solve the following equation system, where 𝜆 is the Lagrange multiplier:

$$\left\{ \begin{aligned} \sum_{i=1}^{n-m} w_{i1}\left( \frac{1}{\lambda^{'}}-z_{i} \right)=0 \\ \sum_{i=1}^{n-m} w_{i2}\frac{z_{i}-\nu^{'}}{{\tau^{'}}^{2}}=0 \\ \sum_{i=1}^{n-m} w_{i2}(\left( z_{i}-\nu^{'} \right)^{2}-{\tau^{'}}^{2})=0 \\ \sum_{i=1}^{n-m} w_{i1}\cdot\frac{1}{p_{1}^{'}}+\lambda=0 \\ \sum_{i=1}^{n-m} w_{i2}\cdot\frac{1}{p_{2}^{'}}+\lambda=0 \\ p_{1}^{'}+p_{2}^{'}=1 \end{aligned} \right..$$

The solution is $\boldsymbol{p}^{t+1}=({\hat{\lambda^{'}}}^{t+1}, {\hat{\nu^{'}}}^{t+1}, {\hat{\tau^{'}}}^{t+1}, {\hat{p_{1}^{'}}}^{t+1}, {\hat{p_{2}^{'}}}^{t+1})$ with

$\left\{ \begin{aligned} {\hat{\lambda^{'}}}^{t+1}={\sum_{i=1}^{n-m} w_{i1}}/{\sum_{i=1}^{n-m} w_{i1}z_{i}} \\ {\hat{\nu^{'}}}^{t+1}={\sum_{i=1}^{n-m} w_{i2}z_{i}}/{\sum_{i=1}^{n-m} w_{i2}} \\ {\hat{\tau^{'}}}^{t+1}=\sqrt{{\sum_{i=1}^{n-m} w_{i2}\left( z_{i}-{\hat{\nu^{'}}}^{t+1} \right)^{2}}/{\sum_{n=1}^{n-m} w_{i2}}} \\ {\hat{p_{1}^{'}}}^{t+1}={\sum_{i=1}^{n-m} w_{i1}}/{(n-m)} \\ {\hat{p_{2}^{'}}}^{t+1}={\sum_{i=1}^{n-m} w_{i2}}/{(n-m)} \end{aligned} \right.$
